# Diffusion of Ions Between Two Solutions Saturated With Respect to Hydroxyapatite: A Possible Mechanism for Subsurface Demineralization of Teeth

**DOI:** 10.6028/jres.115.015

**Published:** 2010-08-01

**Authors:** Laurence C. Chow

**Affiliations:** Paffenbarger Research Center, American Dental Association Foundation, Polymers Division, National Institute of Standards and Technology, Gaithersburg, MD 20899

**Keywords:** calcium phosphate, caries, demineralization, diffusion, dissolution, hydroxyapatite, precipitation

## Abstract

Diffusion-controlled dissolution and precipitation reactions occur in many biological systems and some non-stirred *in vitro* systems. Previous studies have shown that differences in the diffusion rates of the ions involved in a dissolution/precipitation reaction can produce significant effects on the rate and course of the reaction. We report here results of a study that show inter-diffusion of ions between two solutions, both saturated with respect to hydroxyapatite but with dissimilar compositions, resulted in one solution becoming undersaturated and the other supersaturated. A model is proposed that may explain the formation of a mineral-dense layer in the caries process.

## 1. Introduction

Dissolution and precipitation of minerals in many biological systems are believed to be diffusion controlled. One such process that has received substantial interest is demineralization of tooth enamel in the caries process. *In vitro* studies employing a microwell model [1, 2] found that the fluids within tooth enamel or root dentin remained approximately saturated with respect to tooth mineral even when the teeth were exposed to strong demineralization challenges. These results suggest that the rate of dissolution of tooth mineral in the lesion was faster than the rates of transport of acid ions into and/or the mineral ions out of the lesion. Further micro-well studies [3, 4] showed that during the demineralization process a substantial galvani potential difference exists between the fluid within the lesion and the external solution. Such “diffusion potentials” are expected to be present in all processes that involve diffusion of charged species due to differences in inherent ion mobility and the permselective nature of the diffusion barrier. Thus, the subsurface demineralization is largely diffusion-controlled, and the rate of lesion progression may be expected to depend on factors that govern the diffusion process. Studies that employed either a diffusion cell as a bench-scale caries model [5, 6] or a mathematical caries model [7] revealed that interactions between the diffusion and the dissolution processes produced some unexpected results. For a given acid challenge, drastically different lesion solution compositions [5–7] and overall rates of demineralization [7] were obtained as the permselective characteristics of the diffusion barrier were varied.

The present study was aimed at gaining a better understanding of another diffusion related phenomenon: the diffusion of ions between two solutions, both of which were initially saturated with respect to hydroxyapatite (HA) but at different pHs. The solutions were separated by a barrier that prevented mixing of the solutions but allowed diffusion of ions to occur. The diffusion process produced a phenomenon that has not been previously reported: one of the solutions became undersaturated and the other became supersaturated with respect to HA. This process provides a possible mechanism for the formation of a mineral-dense surface layer in a caries lesion and for other pathological decalcification and calcification processes in biological systems.

## 2. Materials and Methods

### 2.1 Solutions Saturated With Respect to Hydroxyapatite (HA)

Two solutions, both saturated with respect to HA but at different pHs, were prepared by dissolving until saturation well-crystallized HA [8] in 1 mmol/L or 20 mmol/L H_3_PO_4_ solution that also contained 30 mmol/L KCl. The pH and the compositions of the two solutions are given in Table 1. It is noted that solution A was a much more concentrated solution in terms of both the calcium ([Ca]) and phosphate ([P]) concentrations (nearly 20 times higher) and the H^+^ activity (about 25 times higher).

### 2.2 Diffusion Cell

**Fig. 1:**
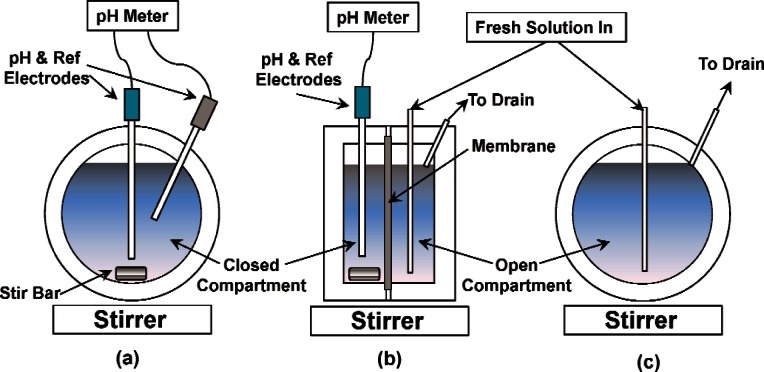
Schematic drawing of the 2-compartment diffusion cell used in the experiments showing side view of the closed compartment (a), front view (b), and side view of the open compartment (c).

The experiments were carried out at (22 ± 1) °C with the use of a two-compartment Plexiglas^®^ diffusion cell (Fig. 1) similar to the cell used in the previous benchscale caries model studies [5, 6]. The dimensions of each cylindrical compartment were 3.1 cm in diameter and 0.25 cm in length. The two compartments were separated by a membrane that was either essentially non-selective (Millipore ultrafiltration membrane PBCC07610, Millipore Corp., Bedford)^1^, cation-permselective (RAI R-4010, Pall RAI Manufacturing Co., Hauppauge, NY), or anion-permselective (RAI R-4035). All membranes were presoaked in 30 mmol/L KCl solution to remove residual acids or bases that might be present. The cell was designed to have a high membrane-area (7.5 cm^2^) to cell volume (1.89 cm^3^) ratio so that the time needed to produce measurable changes in the solution composition brought about by the diffusion process could be reduced.

### 2.3 Experimental Procedure

The two HA-saturated solutions with different pHs were placed in the two compartments of the diffusion cell. In each experiment, the “closed compartment” (CC) (Fig. 1a) was filled with one of the HA-saturated solutions under constant stirring (450 rpm) with the use of a miniature magnetic stirring bar. The “open compartment” (OC) (Fig. 1c) was flushed with the other HA-saturated solution at a constant rate of 0.5 mL/min by means of a metered pump (Sage M375/A, Orion Research, Cambridge, MA). The relatively rapid renewal rate kept the composition of this solution essentially constant while the composition of the solution in the CC continued to change as a result of diffusion of ions between the two solutions.

Two series of experiments were conducted. In experiment 1, the CC was filled with the pH 4.37 HA-saturated solution (solution A), and the OC was flushed with the pH 5.79 HA-saturated solution (solution B). A miniature glass pH electrode (MI-506, Microelectrodes Inc., Londonerry, NH) and a reference electrode (Mi-401, Microelectrodes, Inc.) were placed in the solution in the CC (Fig. 1a) to measure the pH of the solution at various time points. At each of the selected time intervals, a 100 μL aliquot of the CC solution was removed for calcium and phosphate analyses by spectrophotometric methods [10]. The estimated standard uncertainties of the measurements were 0.02 for pH and 2 % for calcium and phosphate analyses. Duplicate experiments were conducted with each of the three types of membranes as the diffusion barrier. A new membrane that had been pre-soaked in the 30 mM KCl solution overnight was used in each run.

In experiment 2, the procedure was the same as that in the first experiment except that the CC was filled with the pH 5.79 HA-saturated solution and the OC was flushed with the pH 4.37 HA-saturated solution.

In this study, no HA solids were present in either solution in order to study the phenomenon resulted solely from the diffusion of ions between the two solutions, both which were initially saturated with respect to HA but had different compositions. As such, the model was not intended to simulate the caries process; rather it was designed to gain insights into driving forces for demineralization and remineralization that could arise simply from the diffusion process.

## 3. Results

**Fig. 2:**
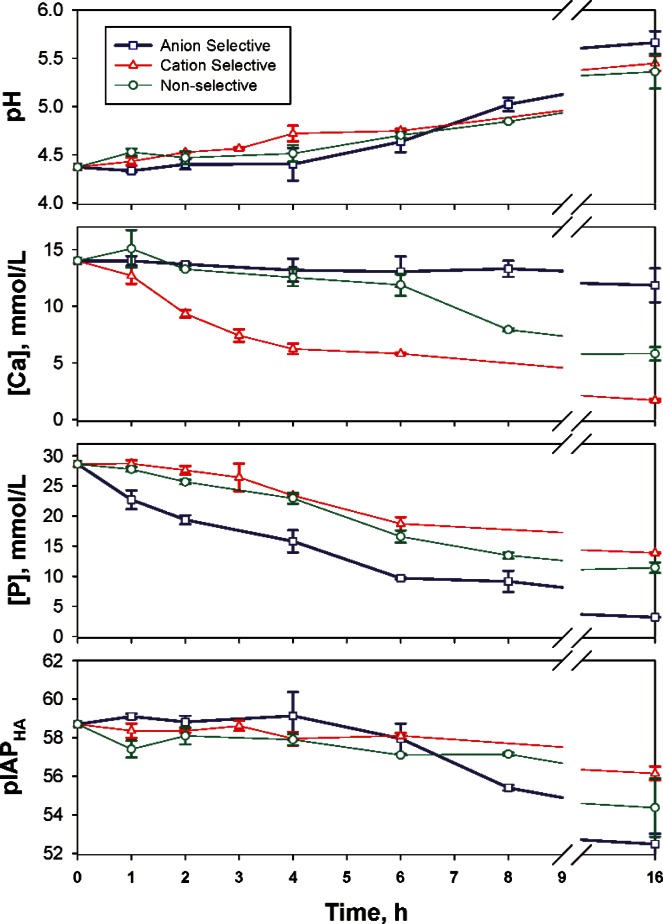
Changes in the composition of the solution in the closed compartment in experiment 1 in which solution A (Table 1) was initially placed in the closed compartment and the open compartment was flushed with solution B. Error bars denote standard deviation (n = 2).

Fig. 2 shows the results of experiment 1. The pH 4.37 HA-saturated solution, placed in the CC at the start of the experiment, was more concentrated (Table 1) than the pH 5.79 HA-saturated solution that flowed through the OC. Consequently, the CC solution lost Ca^2+^, H_2_PO_4_^−^ (the dominant phosphate species) and H^+^ to the OC solution, and both the [Ca] and [P] of the CC solution decreased (Figs. 2b and 2c, respectively) and pH of the solution increased with time (Fig. 2a). The effects of the permselectivity of the membrane can be seen in that the largest decrease in [Ca] was observed with the cation-permselective membrane (Fig. 2b) whereas the largest decrease in [P] was obtained with the anion-permselective membrane (Fig. 2c). The [Ca] and [P] decreased continuously but both were still significantly higher than the corresponding values of the solution in the OC at the end of the 16h period. The pIAP_HA_ (negative logarithm of the ion activity product of HA) values of the solutions, calculated from the [Ca], [P] and pH values and an estimated ionic strength, at the various time points are shown in Fig. 2d. Despite the decreases in [Ca] and [P], all the solutions became supersaturated with respect to HA (lower pIAP_HA_ values). The anion-permselective membrane experiment produced the greatest change in pIAP_HA_ to a value of approximately 53 h by 16 h.

**Fig. 3:**
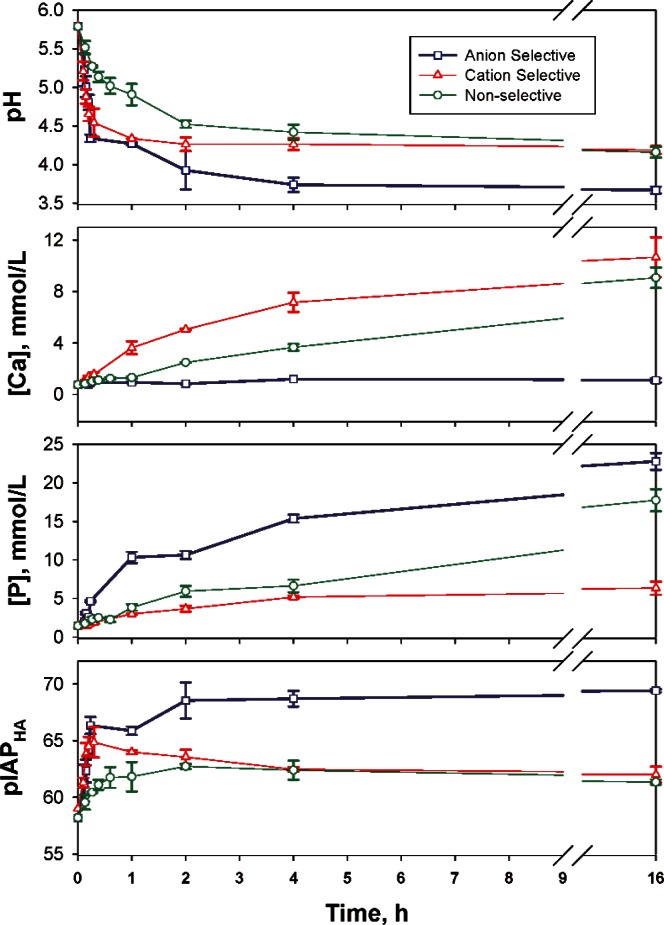
Changes in the composition of the solution in the closed compartment in experiment 2 in which solution B (Table 1) was placed in the closed compartment and the open compartment was flushed with solution A. Error bars denote standard deviation (n = 2).

The results of experiment 2 are shown in Fig. 3. The pH 5.79 solution placed in the CC had lower Ca^2+^, H_2_PO_4_^−^ and H^+^ concentrations than those in the pH 4.37 HA solution that flowed through the OC. This led to increases in both the [Ca] and [P] (Figs. 3b and 3c, respectively) and a decrease in pH of the CC solution with time (Fig. 3a). The effects of membrane permselectivity observed here were similar to those observed in experiment 1, i.e., increase in [Ca] was the largest with the cation-permselective membrane (Fig. 3b), and the increase in [P] was the largest with the anion-permselective membrane (Fig. 3c). Rapid decreases in pH were observed in the first 1 h of the experiment with all membranes; subsequently, the pHs decreased more slowly and reached approximately constant values by 4 h (Fig 3a). At 16 h, the anion-permselective membrane produced the lowest pH value of 3.7, a value that was lower than the pH of the solution in the OC. As a result of the large decreases in pH and in spite of the increases in [Ca] and [P], all solutions became significantly undersaturated with respect to HA (Fig. 3d).

## 4. Discussion

The continuous renewal of the solution in the OC provided a constant background for ion diffusion from or into the solution in the CC. This allowed the effects of ion diffusion on the composition of the CC solution to accumulate. The results described above show that the diffusion of ions between the two solutions, both initially saturated with respect to HA, led to a change in degree of saturation of the CC solution. In experiment 1, the lower pH and more concentrated solution became supersaturated even though it lost Ca^2+^, H_2_PO_4_^−^, and H^+^ ions to the other solution. This appears to be a result of the more rapid diffusion of H^+^ than all other ions [7]. In experiment 2, the rapid gain in H^+^ ion relative to the gains in Ca^2+^ and H_2_PO_4_^−^ ions was also the reason for the higher pH solution to become undersaturated. The limited scope of the present study did not allow a full understanding of the effects of permselectivity of the barrier on the rate of change of HA saturation level in the CC solution. It is clear, however, that as described above the same trend in the changes in HA saturation was observed with all three types of the membranes.

**Fig. 4:**
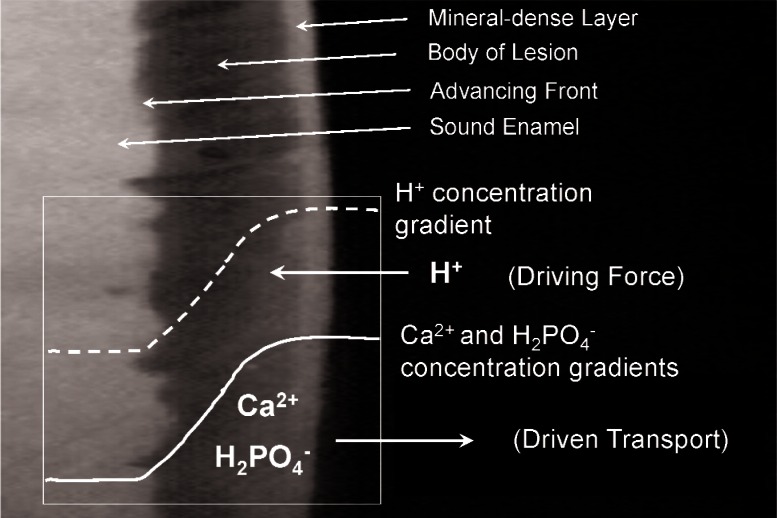
Schematic representation of the driving forces of demineralization in the caries process.

Nearly all human body fluids, e.g., serum, saliva, dentinal fluid, dental plaque fluid, and other extracellular fluids, are slightly supersaturated with respect to HA. However, these fluids do not always have the same ionic compositions. As a result, ion diffusion like the type described here may take place wherever two dissimilar fluids are in contact through a barrier that is permeable to ions. One such example is found in the dental caries process. During the progression of an enamel carious lesion, active demineralization occurs mainly at the “advancing front” of the lesion (Fig. 4). The fluids in this region are connected to the fluids in the sound enamel deeper in the tooth. The latter fluids are connected to the dentinal fluids, which were reported [11] to have [Ca] and [P] of 0.92 mmol/L and 1.10 mmol/L, respectively, and a neutral pH of 7.4. In the opposite direction, the fluids at the advancing front are also in contact with the fluids in the pores in the mineral-dense surface layer through the body of the lesion as the barrier (Fig. 4). The latter fluids, in turn, are connected to the fluids in the plaque. The mineral ion concentrations and the pH of these fluids across a lesion can indeed be quite different. Vogel et al., [12] reported that the fluid in resting plaque had a mean free [Ca] of (0.94 ± 0.29) mmol/L (mean ± standard deviation; n = 15), [P] of (15.1 ± 4.7) mmol/L, and pH of 6.94 ± 0.29. Seven min. after a 1-min. rinse with a sucrose solution (mass fraction of 10 %), the fluids of the challenged plaque had an acidic pH of 5.28 ± 0.3 and mean free [Ca] of (2.7 ± 1.4) mmol/L and [P] of (14.1 ± 4.9) mmol/L. Thus, the plaque fluids in the challenged plaque typically are much more acidic and concentrated than the fluids present at the advancing front of the lesion where mineral dissolution occurs. It was proposed [13] that during lesion progression, the dissolved tooth mineral ions are transported against their concentration gradients (Fig. 4) from the advancing front to the plaque. A process driven by the galvani potential generated by the inward diffusion of H^+^ ions.

The fluids within the lesion and in the plaque would again become approximately saturated with respect to the tooth mineral once the cariogenic condition in the plaque has diminished, i.e., the plaque pH has risen above the critical pH of approximately 5.5 [14]. However, the significant dissimilarities in the pH and mineral ion concentrations between the fluids at different locations in the lesion could bring about the type of diffusion process described in the present study. This can lead to further demineralization at the advancing front (equivalent to the more dilute solution in the present study) and mineral precipitation near the tooth surface (the more concentrated solution).

Several factors are known to promote the formation of a mineral-dense surface layer in caries-like lesions forming processes *in vitro*. The surface layer is preserved by having a surface dissolution inhibitor such as diphosphonate [15] in the demineralizing solution or by the presence of salivary pellicle on the tooth surface [16]. The surface layer may also be preserved by the use of demineralizing solutions that are partially saturated with respect to tooth mineral, contain a gelling compound such as carboxymethyl cellulose, or contain fluoride [17]. The phenomenon described in the present study can be an additional mechanism that could act alone or in combination with the other surface layer preserving factors.

Cussler and Featherstone [18] proposed a model in which the occurrence of simultaneous dissolution and precipitation of mineral is dictated by, among other factors, the valence of the cationic component of the solid. In the present model, which is based on an entirely different principle, the valence of the cation does not seem to be an important factor. As described above, the change in the degree of saturation results primarily from the more rapid transport of H^+^ (OH^−^) ions relative to the mineral ions [7]. Thus, a basic requirement for the simultaneous dissolution-precipitation process is that the solubility of the mineral must increase significantly with decreasing pH. Accordingly, the model would predict that such a process can occur with calcium phosphates, calcium carbonate, calcium hydroxide, etc., but would not occur with calcium sulfate as the solid phase. The only other requirement for this process to occur is that the two solutions with dissimilar compositions are not physically mixed by stirring, convection flow, etc., while the ions are allowed to diffuse across the phase boundary. Such a situation occurs quite commonly in systems such as porous solids, gels, biological tissue, and in other *in vitro* systems where a physical semipermeable barrier is present.

## Figures and Tables

**Table 1:** Compositions of the two hydroxyapatite-saturated solutions at different pHs

Solution	pH	[Ca] mmol/L	[P] mmol/L	[KCl] mmol/L	pIAP_(HA)_
A	4.37	14.0	28.6	30.0	58.7
B	5.79	0.75	1.46	30.0	58.2

Quantities in [ ] are concentrations. pIAP_(HA)_ = −log [(Ca)^5^(PO_4_)^3^(OH)] where quantities in () are ion activities. pKsp (HA) = 58.4 [9].

## Footnotes

1 Certain commercial materials and equipment are identified in this paper to specify the experimental procedure. In no instance does such identification imply recommendation or endorsement by the National Institutes of Health, American Dental Association Foundation, or National Institute of Standards and Technology that the material or equipment identified is necessarily the best available for the purpose.
